# Adaptive Movement Compensation for In Vivo Imaging of Fast Cellular Dynamics within a Moving Tissue

**DOI:** 10.1371/journal.pone.0019928

**Published:** 2011-05-24

**Authors:** Sophie Laffray, Stéphane Pagès, Hugues Dufour, Paul De Koninck, Yves De Koninck, Daniel Côté

**Affiliations:** 1 Centre de Recherche Université Laval Robert-Giffard (CRULRG), Université Laval, Québec, Canada; 2 Centre d'Optique, Photonique et Laser (COPL), Québec, Canada; 3 Département biochimie et de microbiologie, Université Laval, Québec, Canada; 4 Département de psychiatrie et neurosciences, Université Laval, Québec, Canada; 5 Département de physique génie physique et optique, Université Laval, Québec, Canada; University of California, Berkeley, United States of America

## Abstract

In vivo non-linear optical microscopy has been essential to advance our knowledge of how intact biological systems work. It has been particularly enabling to decipher fast spatiotemporal cellular dynamics in neural networks. The power of the technique stems from its optical sectioning capability that in turn also limits its application to essentially immobile tissue. Only tissue not affected by movement or in which movement can be physically constrained can be imaged fast enough to conduct functional studies at high temporal resolution. Here, we show dynamic two-photon Ca^2+^ imaging in the spinal cord of a living rat at millisecond time scale, free of motion artifacts using an optical stabilization system. We describe a fast, non-contact adaptive movement compensation approach, applicable to rough and weakly reflective surfaces, allowing real-time functional imaging from intrinsically moving tissue in live animals. The strategy involves enslaving the position of the microscope objective to that of the tissue surface in real-time through optical monitoring and a closed feedback loop. The performance of the system allows for efficient image locking even in conditions of random or irregular movements.

## Introduction

Advances in understanding complex interactions within neural networks critically depend on our ability to perform functional imaging *in vivo* with subcellular spatial resolution at millisecond time scales [1]–[3]. However, one of the major limitations of high resolution *in vivo* optical microscopy is its extreme sensitivity to tissue movement. Although this is less critical for cortical imaging, since the brain is confined in the skull [4]–[6], this presents a daunting challenge for imaging areas of the nervous system (NS) that are mechanically driven by breathing and cardiac movements (*e.g.*, spinal cord, brain stem). Movements of tens of µm, mainly induced by breathing, dramatically reduce the temporal resolution of measurements because out-of-focus images are unusable. Moreover, any small movement of the tissue, even a few µm, can significantly bias functional measurements with artefactual fluorescence fluctuations.

Three categories of solutions have already been examined: i) elimination of the source of movement, ii) mechanical contention of the tissue being imaged, and iii) computer-assisted methods of post-acquisition image gating. Methods aimed at eliminating the source of movement can be extremely invasive: e.g., interruption of the animal respiration during image acquisition [7], [8]; cardiopulmonary bypass for extracorporal blood oxygenation [9], [10]. Contention methods have limited efficiency, are generally applicable to restricted conditions or regions with limited movement [11], [12]. They can also require dampening local tissue pulsations using agar embedding or applying a cover glass directly on the tissue surface [12], [13]. Such interventions can potentially affect the physiological condition of the tissue and limit duration of observation. Measuring dynamics of an identified structure over hours to days can also be achieved using spatial cues [14], [15] or temporal gating [16] but these strategies cannot be applied to studies of cellular dynamics on millisecond time scale.

As a whole, none of these approaches are fully satisfying, each of them bringing their own constraints which represent a burden when studying the spatiotemporal dynamics of cell ensembles *in vivo*. Thus, new approaches are needed to achieve functional imaging with millisecond resolution in moving tissue with minimal invasiveness.

Here we describe a novel fast adaptive non-contacting device to compensate for movement, implementable on any conventional and commercial two-photon microscope for *in vivo* imaging.

## Results and Discussion

**Figure 1:**
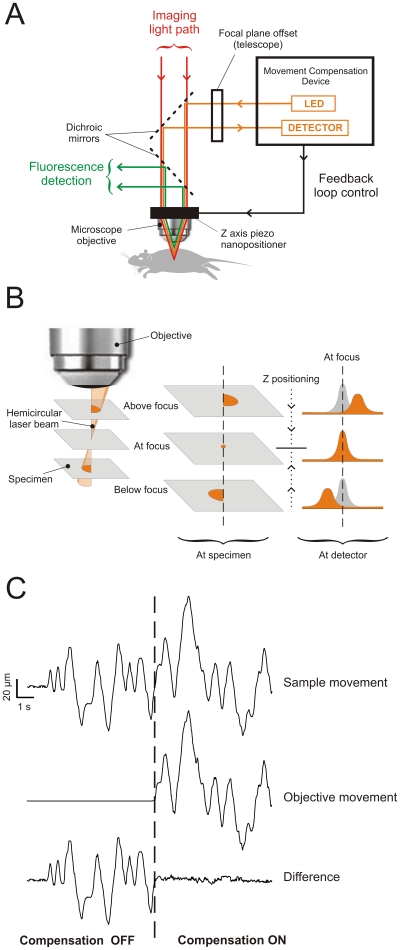
Working principle of the movement compensation device. (A) The illumination and collection paths for imaging (*red trace*) and movement compensation (*orange trace*) are independent. A feedback loop between the movement compensation device and a Z-axis piezo nanopositioner onto which the objective is mounted allows real-time Z movement compensation. Within the tissue, the Z position of the plane to be locked is chosen by adjusting the focal plane offset of the 1∶1 telescope. (B) The illumination for movement compensation is an off-axis hemicircular light beam. When the specimen is at the beam focus, the image of the beam is a point. The image of this point is reflected on the CCD detector as a bell-shaped intensity profile centred at a position corresponding to the in focus plane to be locked. When the plane is above (resp. below) focus, the image is a right-hand (resp. left-hand) hemicircle on the sample and the corresponding projection on the detector is a right (resp. left) shifted bell-shaped intensity profile. (C) Random movement imposed to a sample is compensated by an accurate mirror movement of the piezo onto which the objective is mounted. When the compensation system is activated, the relative movement between the two lies within 2.2 µm (RMS) (*Difference*). Displacements of the piezo and the specimen were independently calibrated.

The device operation is based on the continuous optical monitoring of the position of the tissue and a feedback system to control the position of the objective to maintain constant the distance between the tissue and the objective (Fig. 1A), yielding effective movement compensation. This is achieved by illuminating the sample with an off-axis hemi-circular 785 nm light beam and by measuring the lateral displacement of the reflection onto a linear CCD detector as an indication of Z sample position (Fig. 1B). A control signal derived from the analysis of the reflected pattern is used to adjust a piezo nanopositioner onto which a water immersion objective is mounted, effectively closing the feedback loop (Fig. 1A). The optical paths for monitoring the position of the tissue and the one for imaging are independent (Fig. 1A). The Z position sampling operates at 1 kHz, but the feedback is effectively limited by the nanopositioner which can achieve 250 µm step displacements in 25 ms, which remains orders of magnitude faster than physiological fluctuations (*e.g.*, heart rate and breathing).

Performing non-contacting fast focus control in live tissue to compensate for biological movements is very different from doing drift correction on smooth surfaces [17]. Live tissue imaging brings several important new challenges: i) the surface used to monitor the position is not the same as the imaging plane; ii) the amount of reflected light is very low (orders of magnitude lower than from a glass substrate); iii) surface roughness introduces significant artefacts by coupling transverse and longitudinal movements; iv) fast, real-time feedback is necessary to monitor cellular events that occur at faster time scales than physiological fluctuations (≫1 Hz) (*e.g.*, cellular events on the order of tens of ms in the case of activity in the NS). To address i), an adjustable telescope is added to dynamically modify the distance between the focal plane of the hemicircular beam and the focal plane of the imaging beam. This allows the experimenter to choose, in real-time, imaging planes of interest at different depths within the tissue (Fig. 1A). For ii) and iii), the signal-to-noise ratio (SNR) is optimized using different strategies: first, using a dynamically adapting laser power; second, using a signal processing algorithm involving a rolling window average and digital filtering coupled with a peak fitting procedure and linear interpolation to determine the best current position of the imaging plane of interest. However, low pass filtering introduces poles in closed loop transfer function leading to instability, which in turn requires lowering overall gain. To offset this effect, the predicted Z position for each data point is generated by subtracting the system self motion from the data points before averaging. The correction is then computed based on the best linear fit to extrapolate to current position. This has repeatedly outperformed the conventional proportional-integration-derivation (PID) algorithm in comparison experiments. For iv), the need for real-time correction limits the size of the time window for analysis to several folds shorter than the time needed to acquire a full frame, requiring highly optimized algorithms (for more details of the working principle and algorithm see Fig. S1). Combining the movement compensation strategy with a real-time cross-correlation XY movement correction algorithm [18] yields a full 3D real-time movement compensation during the experiments.

To test the performance of the movement compensation system, i) we imposed a series of calibrated movements to a sample, ii) recorded the dynamics of the objective caused by the feedback control onto the piezo, and iii) assessed the difference between the two. Figure 1C shows an example of random movement imposed on a sample and the resulting movement of the objective driven by the system. The difference between the two movements was around 2.2 µm (RMS) when dynamic focus control was active, demonstrating efficient compensation, even with movement presenting no periodicity. We then tested systematically the performance of the system over a range of amplitudes (5, 35, 50, 100 µm) and frequencies (0.1, 0.5, 1, 2 Hz) on two types of targets: i) a highly reflective, homogeneous mirror target and ii) a highly diffusive heterogeneous biological tissue (rat spinal cord). Over the spectrum of amplitudes and frequencies tested, the effective reduction of the relative movement between the sample and the objective was 93±0.8% with the mirror target and 98±0.8% with the spinal cord sample (Table S1). The remaining amplitude of the uncompensated movement was less than the axial resolution of our video-rate two-photon microscope (approximately 3–5 µm), thus achieving the performance needed for efficient compensation when imaging within moving tissue.

**Figure 2:**
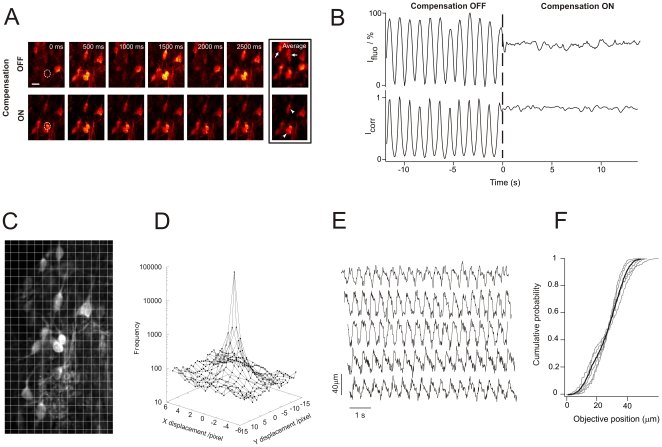
Movement compensation efficiency under *in vivo* conditions. (A) Imaging sequence sampled from a movie recorded at 30 fps of *in vivo* spinal lamina I neurons labelled with a structural dye (Calcein). Each image represents the mean of 5 consecutive raw images, reducing the actual sampling rate to 6 fps. Upper and lower rows were respectively acquired when the compensation was OFF and ON. When it is OFF, the time-stack projection (*Average*) results in an image containing information from planes situated above and below the plane of interest (*arrows*). When it is ON, the time-stack projection results in an image containing information only from the plane of interest, yielding a highly contrasted image (*arrow heads*). Scale bar, 10 µm. (B *upper graph*) Normalized fluorescence intensity time course plotted for one cell in the field of view presented in (a) (dashed ROI) with the system OFF (large intensity fluctuations) and then ON (fluctuations reduced to less than 8% of the initial amplitude). (B *lower graph*) Portion of the time profile of a cross correlation index I_corr_ computed between the first image of Video S1 and each of the following images throughout the entire movie. The presented 25 sec portion is centered around zero, time point where the movement compensation device is turned ON. The ratio of I_corr_ standard deviations with and without movement compensation (92% in this example) was used as a measure of the efficiency of movement compensation in *in vivo* conditions. (C) Extended field of view from which the frames in A are taken. This image is the first frame of the Video S1 (sequence with movement compensation ON). Each frame was divided into a 14×25 grid (each grid element is 13×24 pixels) to perform cross-correlation analysis of pairs of successive images throughout the entire movie. (D) Logarithmic plot of the displacement vector distribution across the image: 70% of the vectors are null while 95% are <2.5 µm. (E) Examples of 5 typical traces of objective movement during compensation. (F) Cumulative probability plot of the objective positions across each of the represented traces (individual traces in grey, average trace in black). The mean objective displacement (5–95%) was 32±6 µm.

We then substantiated the applicability and robustness of the movement compensation device for *in vivo* imaging in the adult rat spinal cord subject to breathing movement. Superficial dorsal horn neurons, from exposed spinal cord of anesthetized adult rats, were labeled *in vivo* by bulk loading of fluorescent dyes. Animals were placed under the laser-scanning video-rate two-photon microscope (Fig. 1A and Materials and Methods). Without any Z movement compensation, the imaging plane changed over time, with cells appearing and disappearing cyclically (Fig. 2A, *upper* and Video S1). In contrast, with the dynamic focus control activated, the imaging plane remained stable over time allowing gap-free imaging (Fig. 2A, *lower* and Video S1). The efficiency of the resulting image stabilization is illustrated by the fact that it yielded sharper, less noisy images when averaging over time (Fig. 2A, *Average*). The reduction in fluorescence fluctuation due to tissue movement is illustrated in figure 2B (*upper graph*). Next, we performed a quantitative measure of the movement compensation efficiency in *in vivo* conditions. First, we computed a cross correlation index (I_corr_) between the first image of Video S1 (movement compensation OFF) and each of the following images throughout the entire movie. The time profile of I_corr_ is presented in Fig. 2B (*lower graph*). Then, for both parts of the graph (with and without movement compensation), I_corr_ standard deviations (σ_Icorr/ON_ and σ_Icorr/OFF_) were computed. We used the expression 1−σ_Icorr/ON_/σ_Icorr/OFF_ as a measure of the efficiency of the movement compensation, which was 92% in this example. Yet, the success of movement compensation is highly dependent on the homogeneity of tissue movement. To ensure that compensation is equally efficient over the entire image, we performed the following analysis: we divided each frame of a video (with movement compensation ON) into a grid (Fig. 2C). Then, we cross-correlated each grid element with its equivalent counterpart (in XY coordinates) in pairs of successive frames across the entire movie. We then derived a displacement vector and plotted the distribution of amplitude of these displacement vectors across the image (Fig. 2D). The analysis revealed that >70% of the vectors are null and 95% of the vector displacements are <2.5 µm, indicating minimal distortion across the image and that the tissue moved uniformly within the chosen length scale. Finally, to illustrate the amplitude of physiological movements that are typically compensated for, we present 5 typical traces of objective movement during compensation (Fig. 2E). For each trace, we built a cumulative probability plot of the positions of the objective (Fig. 2F, grey traces) and extracted the movement amplitude corresponding to probabilities between 5 and 95%: the averaged objective movement amplitude was 32±6 µm. Gap-free imaging is thus achieved without any measure for tissue stabilization, resulting in significantly improved temporal resolution.

**Figure 3:**
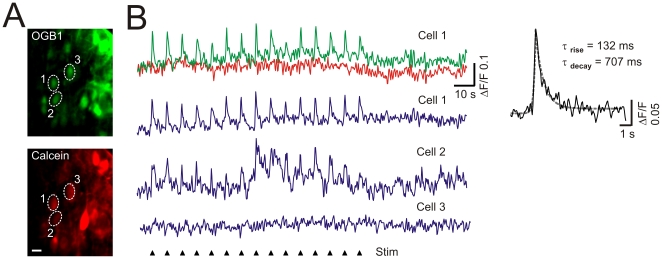
*In vivo* Ca^2+^ imaging of adult spinal lamina I neurons. (A) Spinal lamina I neurons double-labeled with Ca^2+^ dye OGB1 (green) and with the Calcein structural dye (red). ROIs (dashed circles): three selected neurons for (b). Scale bar, 10 µm. (B) The fluorescence intensity fluctuation over time in response to an electrical stimulation of the sciatic nerve (15 pulses of 1 ms, 5 mA at 0.1 Hz) for OGB1 (*green trace, Cell 1*), Calcein (*red trace, Cell 1*) and their subtraction (*blue trace, Cell 1, 2, 3*). *Cell 1* and *2*, but not *Cell 3* displayed Ca^2+^ transients evoked by each stimulus (black arrow heads). Right trace: two exponential functions (one for the rise, one for the decay) were fitted to the Ca^2+^ dynamics (*Cell 1*) averaged over the 15 single trials. τ_decay_ and τ_rise_ were extracted (*Inset*).

We finally tested the performance of the device for *in vivo* functional cellular imaging. For this, we monitored fast Ca^2+^ transients in spinal lamina I neurons of adult rats. Cells were bulk-loaded *in vivo* with 200 µM of Oregon Green BAPTA-1 AM Ca^2+^ dye (Fig. 3B, *OGB1*). As a control of the accuracy of our movement compensation strategy, a structural dye, the Cell Trace Calcein Red Orange, was also loaded at 100 µM together with the OGB1 (Fig. 3B, *Calcein*). Calcein served three purposes: i) as a marker of cell viability, ii) as a reference to measure image stability and iii) to perform correction to enhance signal-to-noise ratio (1). Periodic electrical stimulation of the sensory nerve elicited transient rises in intracellular free Ca^2+^ in specific cells. Each Ca^2+^ response was phase locked with the stimulus and had kinetics comparable to that observed in other *in vivo* imaging experiments [2], [5], [13] (Fig. 3B, *inset*). Some cells also displayed additional Ca^2+^ fluctuations over different time courses. However, no fluctuations in the Calcein signal were observed indicating that i) movement compensation was efficient and ii) the OGB1 fluorescence fluctuations were not due to movement, but rather to different types of Ca^2+^ transients. The gap-free imaging achieved provided sufficient temporal resolution to resolve accurately the fast kinetics of the Ca^2+^ transients (rise time constant = 132 ms±1; decay time constant = 707 ms±42).

In conclusion, the fast adaptive movement compensation system presented here provides a powerful tool for *in vivo* morphological and functional imaging studies of fast cellular dynamics (>10 Hz), even in tissue subjected to large movement (>100 µm). The performance of the system is currently mainly limited by the response time of the piezo nanopositionner: <40 Hz for movements of 250 µm amplitude and up 100 Hz for movements on the order of 10 µm. These limits are likely to be overcome with improvements of the piezo positioning technology or use of non-mechanical focusing strategies [19]. With the performance range dictated by the piezo characteristics, the position sampling system is currently not limiting. Its nominal 1 kHz frequency response is diminished by the need for averaging to improve S/N on rough surfaces, but this could be improved by optimizing the signal processing algorithm. Because the underlying strategy is to adapt the imaging device to movement rather than restricting movement of the tissue, a wide range of applications for live animal studies will benefit from this innovative technology. Furthermore, the non-contact feature makes the approach minimally invasive and thus particularly well-suited for longitudinal studies in delicate or highly reactive tissue. The system could also be adapted for 3D imaging, by implementing a rapid piezo-driven control of the telescope element.

Finally, the system can be exploited for measurements of cellular events taking place within tissue affected by intrinsic movements (e.g. muscle contraction), even in cases where no periodicity occurs.

## Materials and Methods

### Ethics Statement

All experimental procedures were performed in accordance with guidelines from the Canadian Council on Animal Care and the animal protection committee of Laval University (approval 2008019-3).

### Multimodal video-rate imaging system

The video rate microscope has been designed for *in vivo* imaging. The main requirements of such system are: i) the speed of imaging has to be faster than any characteristic movement of the specimen, ii) multimodality to allow for simultaneous acquisition and separation of different signals based on their wavelengths. Typically, an acquisition speed of 30 frames per second while maintaining a relatively high resolution (500×500 scanned points for a 250 µm field of view) must be achieved. The imaging platform has been described in details elsewhere [18]. Briefly, the heart of the system is the fast laser scanning mechanism and its timing circuitry. The approach for the scanning platform is based on the original design of Rajadhyaksha and Webb [20]. It consists in a galvanometer mounted mirror and a spinning polygonal mirror that produce a unidirectional raster scan pattern. The horizontal (or fast) axis is scanned by the spinning polygonal mirror (DT-36-290-025, Lincoln Laser) at a speed of 480 revolutions per second yielding a line scanning rate of approximately 18 kHz. The vertical (or slow) axis scan is performed by the galvanometer-based optical scanner (6240H, Cambridge Technology) coupled to a 15 mm clear aperture silver coated mirror. To obtain the raster scan, the galvanometer mounted mirror is synchronously scanned vertically at 30 Hz.

### Movement compensation device

The device is a highly modified ATF-4 sensor from WDI Inc (Fig. S1). It emits a hemicircular light beam at 785 nm, and an integrated line CCD sensor operating at 7 kHz is used to monitor the position of the reflected beam. An internal processor uses a signal processing algorithm involving a rolling window average (down to 60 Hz) and digital filtering coupled with a peak fitting procedure and linear interpolation to determine the best current position of the imaging plane of interest. The predicted Z position for each data point is generated by subtracting the system self motion from the data points before averaging. The correction is then computed based on the best linear fit to extrapolate to current position. The signal is sent to the nanopositioner for correction.

### Image acquisition and real-time X/Y stabilization processing

Light excitation is provided by a femtosecond Ti-Sapphire laser (Maitai HP, Spectra-Physics, Irvine, CA), operating at 80 MHz with a pulse duration of about 140 fs. The excitation beam is focused onto the sample via a 40× water immersion objective (0.85°NA, Olympus Plan Apochromat). The average power delivered to the samples is typically around 30 mW. An ultrasteep beamsplitter at 45°(LPD01-785RS-25, Semrock, Rochester, NY) is used to combine the laser light used for imaging (λ = 800 nm) with the one used for the movement compensation (λ = 785 nm). The emitted fluorescence is first separated from excitation with a primary dichroic filter (FF735-Di01, Semrock). Then signals arising from the Ca^2+^ dye OGB1 and the structural dye Calcein are separated by a secondary dichroic filter (FF568-Di01, Semrock), filtered respectively with emission filters 525/50 (FF01-525/50, Semrock) and 620/52 (FF568-Di01, Semrock) and finally focussed on two photomultiplier tubes (R3896, Hamamatsu). The analog signal from each detector and the synchronization signals, VSYNC and HSYNC, are sent to a video acquisition board (Snapper-24, Active Silicon). A typical pixel clock, internally generated by the frame grabber board, of 10 MHz is used. This corresponds to 100 ns of pixel dwell time. The software used for acquisition, written in-house, allows for real-time XY movement correction that enables in-plane stabilization with a real-time visual feedback to the user [18]. The real-time XY correction is based on the cross-correlation between two successive frames that gives an instantaneous displacement vector used to correct the position of the first frame with respect to the second one.

### Measurement of the movement compensation accuracy

To test the performances of the system, we imposed a series of movements to a sample (either highly reflective or highly diffusive) mounted on a micromanipulator (MPC-385, Sutter) and monitored simultaneously the movement of the objective and the one of the manipulator. For the former, the piezo controller (E-665, PI) brings a real-time readout of the exact position of the nanopositioner onto which the objective is mounted. For the latter, a glass pipette with a thin tip (∼1 µm) was also fixed to the micromanipulator and imaged at video-rate with a CCD camera for which the pixel size is known. This ensures an independent calibration of the micromanipulator movement.

### Animal surgery for *in vivo* experiments and *ex-vivo* spinal cord preparation

Adult male Wistar rats (250–300 g body weight) were deeply anaesthetized with 4 vol % isoflurane applied through an anaesthesia mask or with urethane (1.5 g/kg i.p.). For experiments conducted under isoflurane, anesthesia was maintained using 1.5 vol % isoflurane. Core temperature was kept at 37.5±2°C with a feedback-controlled heating blanket. The level of anesthesia and the cardiopulmonary health were continuously monitored with a Mouse Ox non-invasive sensor clip (Starr Life Science, Oakmont, PA). Note that movement compensation was achieved with the movement compensation device in both conditions of anaesthesia, even though complete immobilisation of the spinal cord appears difficult to achieve under urethane anaesthesia [11].

The lumbar segments L4–L6 were exposed by laminectomy. A portable stereotaxic frame with two adjustable clamps for vertebral fixation, rostral and caudal to the exposed section, was custom made to fit on a micromanipulator (MP-125, Sutter Instrument, Novato, CA), under the microscope (see above). An agar pool was formed around the exposed spinal segments and filled with artificial cerebrospinal fluid (ACSF). The dura and pia maters were incised, and the lamina I of the dorsal horn of the spinal cord was directly accessed by gently lifting the roots. After the imaging experiment, animals were decapitated under deep anaesthesia. For experiment on the tissue target, a piece of spinal cord was isolated and fixed overnight in 4% PFA, rinsed several times in 0.1 M phosphate buffer.

### *In vivo* dye loading of spinal cord neurons

We adapted the bulk-loading method of fluorescent indicator previously published by Garaschuk et al. [21]. Briefly, a 2 mM stock of the Ca^2+^ dye Oregon Green BAPTA-1 AM (OGB1 AM, Invitrogen, Eugene, OG) in DMSO +20% pluronic (made fresh for each experiment) and a 1.3 mM stock of the structural dye Cell Trace Calcein Red Orange (Calcein, Invitrogen) were respectively diluted 10 and 13 fold in a HEPES-buffered solution composed of the following (in mM): 126 NaCl, 10 HEPES, 2.5 KCl, 2 MgCl2, 2 CaCl2, and 10 glucose (pH 7.4). 200 nl of the mixture was pressure ejected into the superficial laminae (100 µm in depth) of the exposed spinal cord via a glass pipette (resistance around 300 kΩ) connected to a nanoinjector (Micro 4, WPI) at the rate of 50 nl/min. After 30 min of de-esterification of the AM dye, the animal was placed under the video-rate two-photon microscope for imaging.

### *In vivo* electrical stimulations of the sciatic nerve for Ca^2+^ imaging of spinal cord neurons

For this experiment, animals were paralysed with pancuronium bromide and ventilated (Inspira asv, Harvard Apparatus). The left sciatic nerve was exposed during the surgery and a double silver hook electrode was implanted to allow for electrical stimulations of this nerve (15 pulses of 1 ms-duration, 5 mA at 0.1 Hz synchronized with imaging sequence).

### Data analysis for Ca^2+^ transient dynamics

Analysis was performed using custom macros in Image J (National Institutes of Health, Bethesda, MD; http://rsb.info.nih.gov/ij). Regions of interest (ROIs) were manually defined to closely approximate the outline of the structure of interest (neural cell bodies). The fluorescence intensity time courses were measured for both dyes (OGB1 and Calcein, respectively green and red traces Fig. 3 B), and expressed as ΔF/F = (F−F _basal_)/F _basal_. F is the fluorescence at any time point, and F _basal_ the baseline fluorescence acquired at the very beginning of an experiment, before any stimulation. ΔF/F for a given frame is calculated on the mean of all pixels in a ROI. ΔF/F calculated for Calcein can be subtracted to ΔF/F calculated for OGB1 to improve the SNR (Fig. 3 B, blue traces).

To evaluate the kinetics of individual Ca^2+^ responses, the τ_rise_ and the τ_decay_ were extracted from the fit of two exponential functions (one for the rise, one for the decay) to the average of the 15 responses of Cell 1.

## Supporting Information

Figure S1 **Principle of half-aperture method of distance to focus measurement using a hemicircular beam.** The transverse shift (X_in_) in the detector image plane of the centre of gravity (COG) of the bell shaped beam profile from the initial position measured at best focal plane (X_make0_) is in first approximation proportional to the distance from focus measured along the optical axis (X_in_). The measured distance to focus is used as a measurement error signal in a closed loop control system which attempts to minimize the error. The closed loop control is a modified variant of the industry standard PID control with only K_D_ (differential) and K_P_ (proportional) gains used. Modifications were made to address the problem of measurement method that proved to be susceptible to the local variation of tissue density at the point of focus of the ATF4 illumination laser. The biological tissue is reflecting only small portions of the incident light and has a tendency to scatter. This phenomenon is causing the light measured by the detector to be received from a substantially larger surface area (as compared to the diffraction limited spot at focus) and from a region of substantial depth. This, in turn, results not only in a broader beam profile, but also makes the COG position dependent on a local tissue structure. Because in live animal experiments the cyclical movement has both Z and X components, the observed position includes a significant error that, if left not compensated, leads to vibrations. To increase the signal-to-noise ratio, the ATF4 control loop is optimized using different strategies: first, using a dynamically adapting laser power; second, using a signal processing algorithm involving a limiter and rolling window average of N_Avg_ samples and digital filtering coupled with a peak fitting procedure to determine the best current position of the imaging plane of interest. However, low pass filtering of N_Avg_ samples introduces poles in the closed loop transfer function leading to instability, which, in turn, requires lowering the overall proportional gain K_P_. To offset this effect, the predicted position Z_Abs Pred_ for each data point is generated by decoupling the system self motion from the measured position before averaging. The correction is then computed in a linear predictor based on the best linear fit and is extrapolated to a current position. The difference in linearized correction and current position serves as a differential component of the control loop and is sent to the output with the gain K_D_. The ATF4 control loop takes advantage of large signal oversampling, since measurements were available at 1 kHz rate, while the highest frequency component in the signal was estimated at 15 Hz. It should also be noted that to correctly provide the Z_Abs Pred_ position, the system needs to have N_DS_ delay identified, which corresponds to the difference in time between induced motion and observed results. The limiter prevents signal excursions that are too rapid and could not result from biological movements. The rolling window average signal Z_Abs Avg_ is used as reference to center the limiter acceptance band. Because of the use of a piezo actuator, the absolute Z position Z_Abs Out_ (rather than Z delta correction) was necessary as a control output.
(TIF)

Table S1 **Residual movement amplitude (µm) with movement compensation.** Residual movement amplitude (in µm), not compensated by the device, over a range of calibrated movement in terms of amplitude and frequencies applied to two types of targets: one highly reflective and homogeneous (a mirror) and one highly diffusive and heterogeneous (a tissue). Data are means ± SEM of 3 experiments per condition, 10 measurements per experiment. There were no significant differences in mean residual movement amplitudes obtained with the mirror *versus* tissue targets (two-way ANOVA; *P*>0.05).
(DOC)

Video S1 **Movie illustrating movement compensation efficiency under** ***in vivo*** **conditions.** Complete sequence from experiment shown in Fig. 2A. The breathing of the living animal under anesthesia induces a large amplitude movement, resulting in spinal neurons appearing and disappearing cyclically. When the movement compensation is ON, the imaging plane is locked and stable over time. The raw images were corrected for background noise. Scale bar, 10 µm.
(AVI)
