# Supplementary figures and images for: Adaptive Movement Compensation for In Vivo Imaging of Fast Cellular Dynamics within a Moving Tissue
Source: PLoS One. 2011 May 24;6(5):e19928. doi: 10.1371/journal.pone.0019928 (PMC3101223; doi:10.1371/journal.pone.0019928)

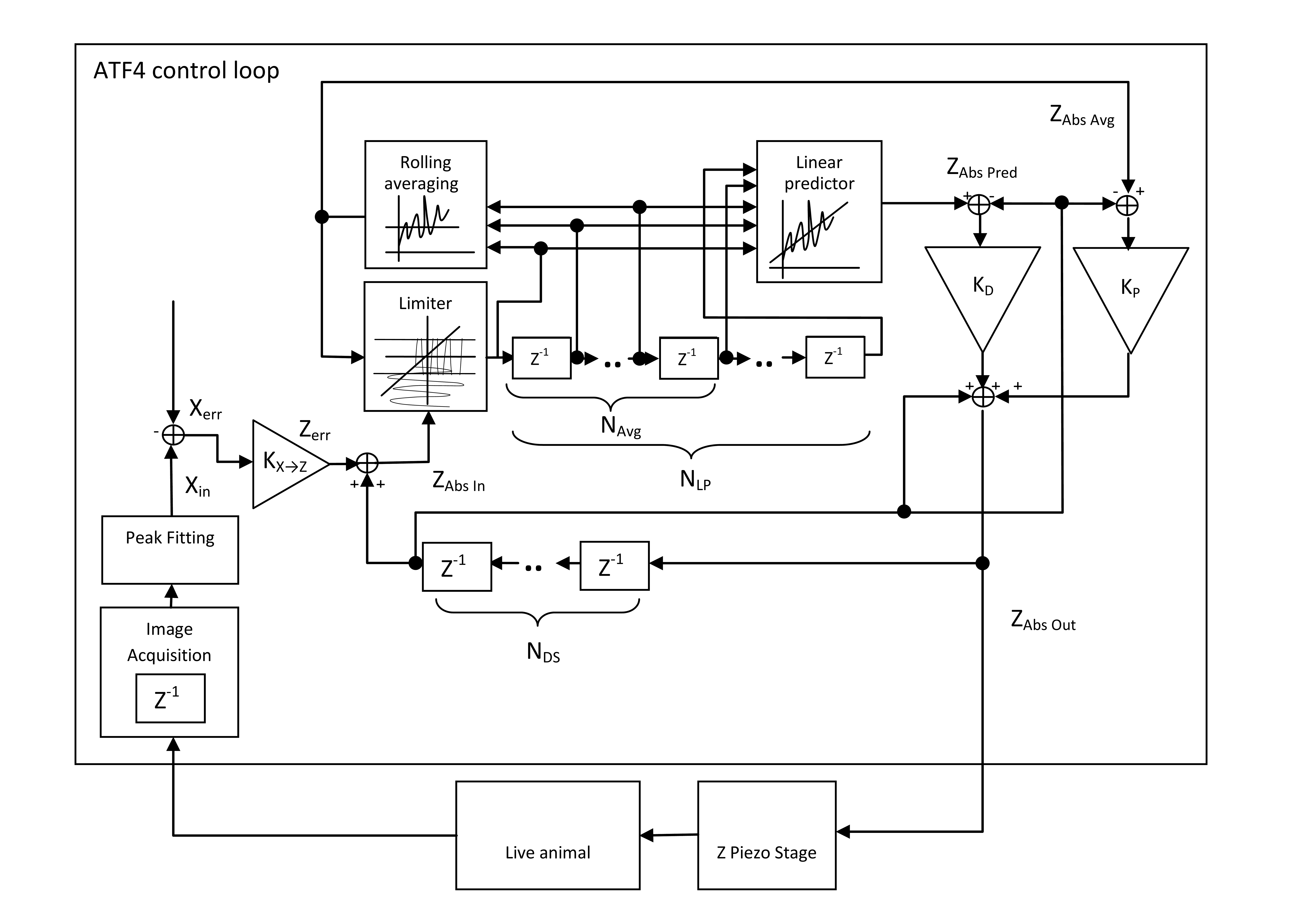

Supplement: Figure S1 — Principle of half-aperture method of distance to focus measurement using a hemicircular beam. The transverse shift (Xin) in the detector image plane of the centre of gravity (COG) of the bell shaped beam profile from the initial position measured at best focal plane (Xmake0) is in first approximation proportional to the distance from focus measured along the optical axis (Xin). The measured distance to focus is used as a measurement error signal in a closed loop control system which attempts to minimize the error. The closed loop control is a modified variant of the industry standard PID control with only KD (differential) and KP (proportional) gains used. Modifications were made to address the problem of measurement method that proved to be susceptible to the local variation of tissue density at the point of focus of the ATF4 illumination laser. The biological tissue is reflecting only small portions of the incident light and has a tendency to scatter. This phenomenon is causing the light measured by the detector to be received from a substantially larger surface area (as compared to the diffraction limited spot at focus) and from a region of substantial depth. This, in turn, results not only in a broader beam profile, but also makes the COG position dependent on a local tissue structure. Because in live animal experiments the cyclical movement has both Z and X components, the observed position includes a significant error that, if left not compensated, leads to vibrations. To increase the signal-to-noise ratio, the ATF4 control loop is optimized using different strategies: first, using a dynamically adapting laser power; second, using a signal processing algorithm involving a limiter and rolling window average of NAvg samples and digital filtering coupled with a peak fitting procedure to determine the best current position of the imaging plane of interest. However, low pass filtering of NAvg samples introduces poles in the closed loop transfer function leading to [file pone.0019928.s001.tif]
