# Supplementary material for: Adaptive Movement Compensation for In Vivo Imaging of Fast Cellular Dynamics within a Moving Tissue
Source: PLoS One. 2011 May 24;6(5):e19928. doi: 10.1371/journal.pone.0019928 (PMC3101223; doi:10.1371/journal.pone.0019928)
Supplement: Table S1 — Residual movement amplitude (µm) with movement compensation. Residual movement amplitude (in µm), not compensated by the device, over a range of calibrated movement in terms of amplitude and frequencies applied to two types of targets: one highly reflective and homogeneous (a mirror) and one highly diffusive and heterogeneous (a tissue). Data are means ± SEM of 3 experiments per condition, 10 measurements per experiment. There were no significant differences in mean residual movement amplitudes obtained with the mirror versus tissue targets (two-way ANOVA; P>0.05). (DOC) [file pone.0019928.s002.doc]

**SUPPORTING TABLE S1: Residual movement amplitude (μm) with movement compensation**

|  | **Tissue target** | | | | **Mirror target** | | | |  |  |  |
| --- | --- | --- | --- | --- | --- | --- | --- | --- | --- | --- | --- |
|  | **5 µm** | **35 µm** | **50 µm** | **100 µm** | **5 µm** | **35 µm** | **50 µm** | **100 µm** |  | | |
| **0.1 Hz** | 0.3 ± 0.1 | 2.2 ± 0.6 | 3.1 ± 0.1 | 5.5 ± 0.5 | 1.8 ± 0.6 | 2.1 ± 2.5 | 0.0 ± 1.8 | 2.4 ± 2.8 |  | | |
| **0.5 Hz** | 0.6 ± 0.0 | 1.8 ± 0.4 | 2.8 ± 0.5 | 5.3 ± 0.3 | 0.2 ± 0.8 | 0.5 ± 0. 1 | 2.3 ± 1.4 | 5.2 ± 3.2 |  | | |
| **1 Hz** | 0.6 ± 0.2 | 1.9 ± 0.3 | 2.8 ± 0.4 | 4.9 ± 0.3 | 0.1 ± 0.3 | 1.4 ± 2.4 | 1.9 ± 3.1 | 0.5 ± 1.5 |  | | |
| **2 Hz** | 0.7 ± 0.3 | 2.7 ± 0.3 | 2.5 ± 0.3 | 4.8 ± 0.2 | 0.2 ± 1.6 | 1.5 ± 1.5 | 2.8 ± 1.1 | 1.6 ± 4.4 |  | | |

Residual movement amplitude (in μm), not compensated by the device, over a range of calibrated movement in terms of amplitude and frequencies applied to two types of targets: one highly reflective and homogeneous (a mirror) and one highly diffusive and heterogeneous (a tissue). Data are means ± SEM of 3 experiments per condition, 10 measurements per experiment. There were no significant differences in the mean residual movement amplitudes obtained with the mirror *versus* tissue targets (two-way ANOVA; *P*>0.05).
